# The association between age at menarche and later risk of gestational diabetes is mediated by insulin resistance

**DOI:** 10.1007/s00592-018-1162-7

**Published:** 2018-05-22

**Authors:** Clive J. Petry, Ken K. Ong, Ieuan A. Hughes, Carlo L. Acerini, David B. Dunger

**Affiliations:** 1 Department of Paediatrics, University of Cambridge, Cambridge Biomedical Campus, Hills Road, Box 116, Cambridge, CB2 0QQ UK; 2 Medical Research Council Epidemiology Unit, University of Cambridge, Cambridge, CB2 0QQ UK; 3 The Institute of Metabolic Science, University of Cambridge, Cambridge, CB2 0QQ UK

**Keywords:** Puberty, Pregnancy, Insulin sensitivity, Adiposity, PAPP-A

## Abstract

**Aims:**

Associations have been reported between age at menarche and the later risk of gestational diabetes. However, it is not known whether these associations reflect differences in insulin sensitivity and/or pancreatic β-cell function in pregnancy.

**Methods:**

We examined this question in women enrolled in the prospective Cambridge Baby Growth Study who recalled their age at menarche in questionnaires during pregnancy. Polynomial logistic and linear regression models were used to relate menarche timing to the risk of gestational diabetes, both unadjusted and adjusted for the Homeostasis Model Assessments of insulin resistance (HOMA IR) and pancreatic β-cell function (HOMA B) at week 28 of pregnancy.

**Results:**

Age at menarche showed a U-shaped association with gestational diabetes risk (linear term: *p* = 9.5 × 10^−4^; quadratic term: *p* = 1.0 × 10^−3^; *n* = 889; overall model *p* = 8.1 × 10^−3^). Age at menarche showed a negative linear association with insulin resistance (HOMA IR: *β* = −0.13, *p* = 5.2 × 10^−4^, *n* = 771), which explained the relationship between age at menarche and gestational diabetes risk (adjusted linear term going from *p* = 0.03–0.08; adjusted quadratic term going from *p* = 0.04–0.08; *n* = 771). Age at menarche also showed a negative linear association with β-cell function (HOMA B: *β* = −0.11, *p* = 2.8 × 10^−3^, *n* = 771) but this did not attenuate the relationship between age at menarche and gestational diabetes (adjusted linear term *p* = 0.02; adjusted quadratic term *p* = 0.03, *n* = 771).

**Conclusions:**

These results suggest that the associations between age at menarche and risk of gestational diabetes and raised pregnancy glucose concentrations may be mediated by insulin resistance.

**Electronic supplementary material:**

The online version of this article (10.1007/s00592-018-1162-7) contains supplementary material, which is available to authorized users.

## Introduction

Many settings have observed secular trends towards a lowering of average age at menarche (AAM) in the last 50 years, coincident with higher levels of nutrition [1]. The AAM appears to be dependent on both genetic and environmental factors [2]. The trend towards its lowering is thought to be related to the improved socioeconomic conditions and the higher prevalence of childhood obesity [3]. Possibly through reflecting differences in the reproductive hormonal milieu, changes in AAM may be important since it appears to be related to health in adult life through associations with risk of adult obesity [4], type 2 diabetes [5], asthma [6], endometriosis [7], breast cancer [8] and death (by all causes [9]).

Not surprisingly given the strong links between type 2 diabetes and gestational diabetes (GDM) and the association between AAM and type 2 diabetes, recently AAM has also been found to be associated with the future risk of GDM in pregnancy [10–13]. As long ago as 1975 it was suggested that early menarche may be linked to GDM [14]. In each of the studies the highest risk of GDM was in women with the earliest AAM. However, this association has not been consistently found across all populations [15]. In the studies where an association between AAM and risk of future GDM was observed, no attempt was made to relate AAM to the principal mechanisms of glucose regulation: insulin sensitivity and secretion in pregnancy. In the present analysis we therefore tested whether AAM was associated with future GDM risk and raised glucose concentrations in pregnancy, and how such associations relate to changes in indices of insulin sensitivity and pancreatic β-cell function/insulin secretion in the Cambridge Baby Growth Study. Early pregnancy-associated plasma protein A (PAPP-A) concentrations were also assessed in relation to AAM since these are potential biomarkers of GDM risk [16] that appear to relate to changes in insulin sensitivity in pregnancy [17].

## Materials and methods

### Cohort

The prospective Cambridge Baby Growth Study recruited 2,229 women (and consequently their partners and babies) attending ultrasound clinics during early pregnancy at the Rosie Maternity Hospital, Cambridge, United Kingdom, between 2001 and 2009 [17, 18]. All study participants were over 16 years of age. At around 15 weeks of gestation (range 12–18 weeks) the mothers had a blood sample collected for the measurement of serum PAPP-A [17]. At around 28 weeks of gestation the mothers without known diabetes underwent a 75 g oral glucose tolerance test (OGTT) after fasting overnight. Venous blood was collected just prior to and 60 min. (and 120 min. after 2007) after the consumption of the glucose load for the measurement of plasma glucose, insulin and C-peptide concentrations. Capillary blood glucose measurements were made at 0, 30, 60, 90, and 120 min. using an Abbott Freestyle Mini (Abbott Diagnostics, Maidenhead, UK). GDM, with a prevalence of 10.2% in the whole cohort, was defined here using the International Association of Diabetes in Pregnancy Study Groups thresholds [19] using the 0 and 60 min. venous plasma glucose concentrations as described [20].

Each study participant was given a printed questionnaire at recruitment to fill in and return once the pregnancy was completed. One of the questions asked “What age were you when you had your first period?” which the study participants tended to answer in terms of whole years. A total of 1273 women (57.1%) filled in and returned their questionnaires.

### Ethics

The Cambridge Baby Growth Study was approved by the local ethics committee, Addenbrooke’s Hospital, Cambridge, United Kingdom. All procedures followed were in accordance with the institutional guidelines and therefore the ethical standards as laid down in the 1964 Declaration of Helsinki and its later amendments. Written informed consent was obtained from all the study participants prior to their inclusion in the study.

### Laboratory measurements

Serum PAPP-A was measured using a time-resolved fluorescence immunoassay as described previously [17]. Venous plasma glucose concentrations were measured using a routine glucose oxidase-based method. Plasma C-peptide and insulin concentrations were measured using Diagnostic System Laboratories (London, UK) ELISAs run according to the manufacturer’s instructions. The sensitivity of the C-peptide assay was 0.004 nmol/L. Its intra-assay CV was 2.4% at 3.1 nmol/L and its equivalent inter-assay CV was 2.7%. The sensitivity of the insulin assay was 2 pmol/L. Its intra-assay CV was 4.4% at 72 pmol/L and its equivalent inter-assay CV was 8.7%.

### Calculations

The body mass index (BMI) was calculated as the self-reported pre-pregnancy body weight divided by the height squared. Insulin sensitivity and pancreatic β-cell function (insulin secretion) were estimated using the Homeostasis Model Assessment (HOMA), calculated using the week 28 circulating glucose and C-peptide concentrations using the online HOMA calculator [21]. Insulin secretion was quantified as the C-peptidogenic index, calculated as (C-peptide 60 min—C-peptide 0 min)/(glucose 60 min—glucose 0 min). The insulin secretion for the given insulin sensitivity was assessed in terms of the C-peptide disposition index, calculated as the C-peptidogenic index divided by the fasting C-peptide concentration. Equivalent indices were calculated using insulin instead of C-peptide concentrations but C-peptide-derived variables were used in the statistical models so that they were not affected by hepatic insulin extraction. The areas under the OGTT capillary whole blood glucose curves (AUC) were calculated using the trapezoid rule.

### Statistical analyses

The association between AAM and GDM was analysed using logistic regression, both unadjusted and adjusted for covariates. Polynomial logistic regression was also used to test for a non-linear association as reported by Li et al. [12] and also apparent in other studies [11, 13, 15]. In our analysis the GDM risk was fitted using Stata’s qfit function, having already tested whether or not there was a linear relationship with AAM. We then tested which additional metabolic parameters attenuated the linear and quadratic components of the GDM risk, using just a subset of the samples for whom we had HOMA data available.

The Shapiro–Wilk test was used to test for normality. Unless otherwise stated, all other data are presented as means (95% confidence intervals). Where regression coefficients (*β*) are shown in analyses of continuous variables they are standardised throughout. Statistical analyses were performed using Stata 13 (StataCorp LP, College Station, Texas, U.S.A.). *p* < 0.05 was considered statistically significant throughout.

## Results

### Characteristics of the study participants

In this cohort, 96.9% of the babies were of white ethnicity, 0.8% were of mixed race, 0.6% were black (African or Caribbean), 0.8% were East-Asian, and 0.9% were Indo–Asian. Only those mothers that returned their completed questionnaires were included in this analysis. Of these the mean AAM was 12.9 years and the median was 13 (interquartile range 12–14) years. The clinical characteristics of those women that were included and those that were excluded from the present analysis due to non-return of their questionnaires are shown in Supplementary Table 1 (Online Resource 1). The unadjusted birth weights were higher in those included in the present analysis (alongside a 2 day older gestational age at birth), although the difference in birth weight disappeared when adjustment was made for gestational age. There were proportionally fewer women that smoked during pregnancy amongst those that were included in this analysis. The distribution of the AAM was normal (Shapiro–Wilk *p* = 0.9).

### Association between age at menarche and gestational diabetes

Clinical characteristics of those study participants who developed GDM (9.8%) and those that did not are shown in Supplementary Table 2 (Online Resource 1). There was no significant difference in AAM between those that developed GDM and those that did not. In linear models with AAM as the independent variable there was no association between AAM and later GDM risk [odds ratio (OR) 0.97 (0.83, 1.13) per year, *p* = 0.7, *n* = 889]. However, in quadratic models there was a significant U-shaped association between AAM and GDM at week 28 [linear term OR 0.08 (0.02, 0.36) per year, *p* = 9.5 × 10^−4^; quadratic term OR 1.10 (1.04, 1.17), *p* = 1.0 × 10^−3^; *n* = 889; overall model pseudo r^2^ = 1.7% and *p* = 8.1 × 10^−3^]. Relative risks for individual AAM categories are shown in Table 1. Restricting the analyses to the samples for which we had pre-pregnancy BMI data; there was still a U-shaped association between AAM and GDM risk [linear term OR 0.08 (0.02–0.40) per year, *p* = 2.0 × 10^−3^; quadratic term OR 1.10 (1.03–1.17), *p* = 2.4 × 10^−^^3^; *n* = 798]. AAM showed a negative linear relationship with pre-pregnancy BMI (*β* = −0.22, *p* = 6.0 × 10^−10^, *n* = 798) but addition of pregnancy BMI did not attenuate either the linear or quadratic terms of the association between AAM and GDM [adjusted linear term OR 0.08 (0.02, 0.41) per year, *p* = 2.5 × 10^−3^; quadratic term OR 1.10 (1.04, 1.18), *p* = 2.1 × 10^−3^; *n* = 798].

**Table 1:** The relative risks of GDM in pregnancy in the Cambridge Baby Growth Study for groups stratified by AAM

Age at menarche (years)	Gestational diabetes [*n* (%)]	Not gestational diabetes [*n* (%)]	Relative risk
8–9.9	3 (33.3)	6 (66.7)	3.7 (1.4, 9.7)
10–11.9	14 (9.6)	132 (90.4)	1.1 (0.6, 1.9)
12–13.9	42 (9.1)	422 (90.9)	Reference
14–15.9	21 (8.8)	217 (91.2)	1.0 (0.6, 1.6)
16–17.9	7 (21.9)	25 (78.1)	2.4 (1.2, 4.9)

AAM showed negative linear but not quadratic associations with both HOMA IR and B (Table 2). We tested whether these insulin traits attenuate the U-shaped association between AAM and GDM risk in a subset of women for which we had full HOMA data available. In this subset, there was still a significant pre-adjustment U-shaped association between age at menarche and GDM, albeit weaker than in the full set of women due to the smaller sample size [linear term OR 0.14 (0.02–0.82) per year, *p* = 0.03, *n* = 771; quadratic term OR 1.08 (1.01–1.16), *p* = 0.04]. This association was attenuated when adjusting for HOMA IR [linear term OR 0.18 (0.03–1.25) per year, *p* = 0.08, *n* = 771; quadratic term OR 1.07 (0.99–1.15), *p* = 0.08], but not when adjusting for HOMA B [linear term OR 0.12 (0.02–0.73) per year, *p* = 0.02, *n* = 771; quadratic term OR 1.08 (1.01–1.16), *p* = 0.03].

**Table 2:** Linear and quadratic associations between AAM and indices derived from the OGTT (and related variables)

OGTT-related variable	*n*	Linear regression		Mixed linear/quadratic regression			
				Linear term		Quadratic term	
		*β*	*p* value	*β*	*p* value	*β*	*p* value
Pre-pregnancy BMI	658	−0.180	3.3 × 10^−6^	0.378	0.5	−0.560	0.3
Maternal height	690	0.209	3.2 × 10^−8^	0.442	0.4	−0.234	0.6
HOMA IR	731	−0.128	5.2 × 10^−4^	−0.925	0.05	0.799	0.09
HOMA B	731	−0.110	2.8 × 10^−3^	−0.499	0.3	0.390	0.4
OGTT 60 min glucose	723	−0.096	0.01	−0.129	0.8	0.034	0.9
Capillary glucose AUC	619	−0.068	0.09	−0.503	0.3	0.436	0.4
C-peptidogenic index	684	−0.002	1.0	−0.669	0.2	0.669	0.2
C-peptide disposition index	684	0.065	0.09	−0.170	0.7	0.235	0.6

In each case the analysis was restricted to those samples where full C-peptide-derived HOMA data were available

### Association between age at menarche and indices from the week 28 oral glucose tolerance test

**Fig. 1:**
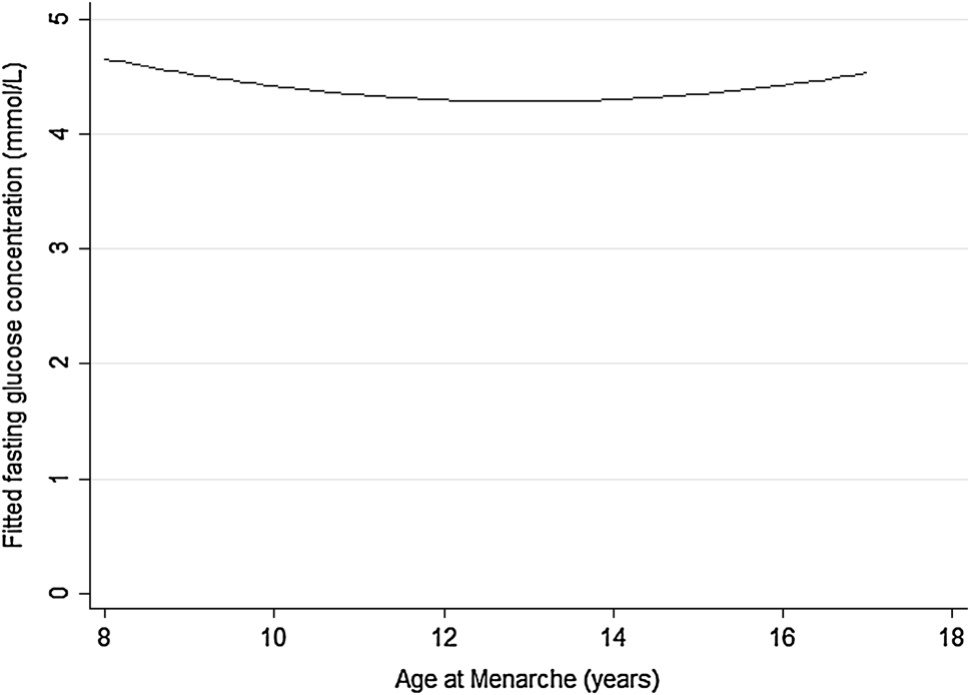
Fitted fasting glucose concentrations in week 28 of pregnancy by AAM in the Cambridge Baby Growth Study

Summary data from the study participants’ OGTTs are shown in Supplementary Table 3 (Online Resource 1). In the full sample set AAM was associated with week 28 fasting glucose concentration in a U-shaped fashion (linear term *β* = −1.049, *p* = 0.01; quadratic term *β* = 1.032, *p* = 0.01; *n* = 889) (Fig. 1). This relationship was non-significant in the sub-sample restricted to those women where HOMA data were available (linear term *β* = −0.785, *p* = 0.08; quadratic term *β* = 0.754, *p* = 0.10; *n* = 771), and was further weakened when adjusted for HOMA IR (linear term *β* = −0.443, *p* = 0.29; quadratic term *β* = 0.465, *p* = 0.27; *n* = 771). In contrast it was strengthened when adjusted for HOMA B (linear term *β* = −0.898, *p* = 0.04; quadratic term *β* =  0.828, *p* = 0.05; *n* = 771). Unlike with the fasting samples, AAM showed negative linear associations with OGTT 60 min. glucose concentrations (Table 2), which persisted after adjusting for HOMA B (*β* = −0.100, *p* = 5.9 × 10^−3^, *n* = 763) but not after adjusting for HOMA IR (*β* = −0.064, *p* = 0.07, *n* = 763).

### Association between age at menarche and serum PAPP-A concentrations earlier in pregnancy

**Fig. 2:**
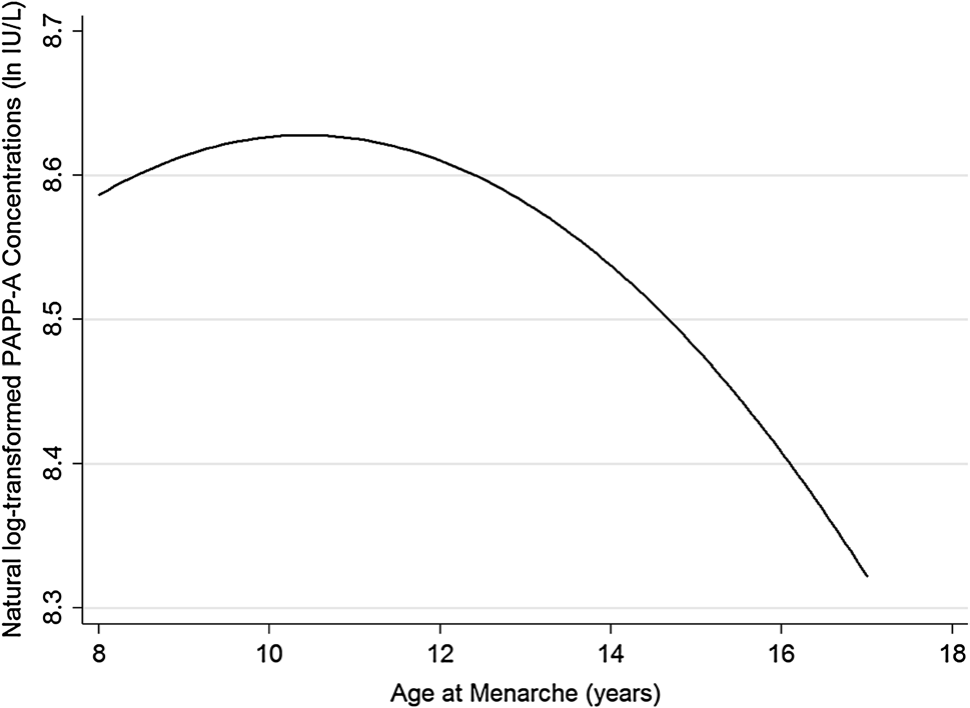
Fitted (natural log-transformed) PAPP-A concentrations around week 15 of pregnancy by AAM in the Cambridge Baby Growth Study

AAM was not linearly related to week 15 serum PAPP-A concentrations (*β* = −0.014, *p* = 0.7, *n* = 501; adjusted for the exact stage of gestation when the blood sample was collected). However, there was an inverse U-shaped association between week 15 serum PAPP-A concentrations and AAM (linear term: *β* = 1.807, *p* = 0.02; quadratic term: *β* = −1.823, *p* = 0.02, *n* = 501; adjusted as above; Fig. 2).

## Discussion

In this analysis we have confirmed associations between AAM and the subsequent higher risks of GDM. Not surprisingly there were also relationships between AAM and raised glucose concentrations (both fasting and stimulated) in pregnancy. Consistent with the findings of Li et al. [12], the relationship between AAM and risk of GDM in our population was U-shaped. This pattern of relationship has also previously been observed between AAM and type 2 diabetes [22], a condition closely related to GDM [23]. Both conditions are thought to result from a combination of insulin resistance and inadequate insulin secretion, with GDM generally just emerging at a younger age because of the additional physiological insulin resistance of pregnancy [23]. Visual inspection of results from other studies investigating links between AAM and GDM risk finds similar relationships to our U-shaped association even if those studies did not formally test for polynomial associations [11–13]. The one published study that failed to find an association between AAM and GDM risk tested only for a linear relationship [15]. However, on inspection of their data there is an apparent U-shaped relationship. Consistent with the study of Schoenaker et al. [11] the relationship that we found in the present study appeared to be independent of BMI. Indeed our data would suggest that the relationship between AAM and GDM risk relates to insulin resistance rather than adiposity.

In all previous studies reporting a significant association, earlier AAM appears to have a larger influence that later AAM on GDM risk [10–13]. Our data are consistent with this pattern. Indeed the earliest AAM group had a nearly fourfold higher risk of GDM relative to the median AAM groups, albeit that this was modelled from only a small number of participants. The small number of participants in this analysis with the earliest AAM may have led to an inflated relative risk of GDM and the true risk for this group may be closer to the lower end of the 95% confidence interval. Indeed for AAM of < 10 years the risk of GDM by meta-analysis of our data combined with that of the other published studies [10–12, 15], relative to those with an AAM of 13 years, had an OR of 1.8 with a 95% confidence interval of 1.6–2.0 (unpublished observation) which is only slightly higher than that reported for an AAM of < 11 years in a recently published meta-analysis in this area, which of course did not include data from the present study [24]. In our study and those other studies showing a U-shaped relationship between AAM and GDM [11, 12, 15] there was also a slight increase in risk for GDM associated with late AAM. This increased risk was not observed in all studies [10, 13], but in our pooled random effects dose response meta-analysis of relevant studies it caused a significant non-linearity term (unpublished observation). The mechanism mediating the association between late AAM and slight increased GDM risk is currently unknown, but could involve changes in the sensitivity or secretion of some of the pregnancy factors described below.

Chen et al. [10] hypothesised that the overall relationship between AAM and GDM risk would be underpinned by changes in concentrations of as yet unspecified hormone(s). Results from the current analysis would suggest that they are likely to be hormones that predominantly alter insulin sensitivity. One such hormone could be PAPP-A since we found an inverse U-shaped relationship between AAM and circulating PAPP-A concentrations around week 15 of pregnancy. Previously we reported that low PAPP-A concentrations at this stage of pregnancy are associated with an increased risk of GDM [17]. PAPP-A concentrations were also associated with reduced third trimester insulin sensitivity, possibly due to the modification of localised IGF bioactivity [25] through its role in cleaving IGF-binding protein 4 [26]. Other hormones that could be involved in mediating the link between AAM and insulin resistant GDM include oestrogens [10] since higher concentrations in women have been linked both to early AAM [27–30] and increased GDM risk (when considered relative to sex hormone binding globulin concentrations [31]). Leptin concentrations have also been associated with both AAM [32] and risk of GDM, independent of adiposity [33, 34]. Finally, in metabolic terms, the link between AAM and GDM risk may also involve triacylglycerols since their circulating concentrations may be related to both AAM [35, 36] and GDM risk [37].

The present analysis has advanced knowledge of the link between AAM and GDM risk in pregnancy by highlighting the mediating role of insulin resistance. Our estimate of pancreatic β-cell function appeared to have a lesser role in AAM-related GDM risk. It did attenuate the OGTT C-peptide disposition index association with AAM, but this is not surprising for an indicator of insulin secretion (relative to its sensitivity). The strengths of our analysis include its prospective nature, the availability of indices related to the glucose-insulin axis in the third trimester of pregnancy from the OGTT, and HOMA modelling using C-peptide rather than insulin concentrations (the former being unaffected by hepatic extraction). Its limitations include the small number of participants in the analysis at the extremes of AAM, which may have led to an inflation of the relative risks of GDM associated with these AAM categories (although there was still a significant relationship evident between AAM and GDM risk, albeit weakened, if the analysis was restricted to women with an AAM between 10 and 16 years; data not shown). This limitation did not substantially affect the overall relationship between AAM and GDM risk as AAM was modelled primarily as a continuous variable. A further limitation is that the ages at menarche were self-reported which can be inaccurate. However, in other studies, self-reported AAM is thought to be moderately accurate in women with higher educational attainment [38] like the majority of women recruited to the Cambridge Baby Growth Study [39]. Finally, as is common with cohort studies that were not designed explicitly to test each different hypothesis in all of the specific studies they are used for, the number of study participants in each of our statistical models varies due to missing data. Whilst this could theoretically introduce a degree of bias into the analyses we have no actual evidence of this and the statistical analyses present a biologically plausible explanation for the association between AAM and GDM risk.

In conclusion this study has confirmed the previously observed U-shaped association between AAM and future GDM risk [10–13] that is independent of pre-pregnancy BMI and therefore adiposity [11]. Our findings add, for the first time, that insulin resistance appears to mediate this relationship. Future studies should continue to investigate the possible hormonal mechanism(s) linking age at menarche to insulin resistance and subsequent risk of GDM.

### Data availability

The datasets generated during and/or analysed during the current study are available here: 10.17863/CAM.18259.

## Electronic supplementary material

Below is the link to the electronic supplementary material.

Supplementary material 1 (DOCX 17 KB)
