# Supplementary material for: The association between age at menarche and later risk of gestational diabetes is mediated by insulin resistance
Source: Acta Diabetol. 2018 May 22;55(8):853–9. doi: 10.1007/s00592-018-1162-7 (PMC6060956; doi:10.1007/s00592-018-1162-7)
Supplement: Supplementary file 1 — Supplementary material 1 (DOCX 17 KB) [file 592_2018_1162_MOESM1_ESM.docx]

Supplementary Tables for

**The Association between Age at Menarche and Later Risk of**

**Gestational Diabetes is mediated by Insulin Resistance**

Clive J. Petry^1^, Ken K. Ong^1-3^, Ieuan A. Hughes^1^, Carlo L. Acerini^1^, David B. Dunger^1, 3^.

^1^Department of Paediatrics, University of Cambridge, Cambridge CB2 0QQ, U.K.

^2^Medical Research Council Epidemiology Unit, University of Cambridge, Cambridge CB2 0QQ, U.K.

^3^The Institute of Metabolic Science, University of Cambridge, Cambridge CB2 0QQ, U.K.

Corresponding author: Dr. Clive Petry, E-mail: [cjp1002@cam.ac.uk](mailto:cjp1002@cam.ac.uk)

**Supplementary Table 1** Characteristics of the Cambridge Baby Growth Study sample included in this analysis, and those who were excluded due to lack of prenatal questionnaire data.

| **Characteristic** | **Included Participants** | **Excluded Participants** | **p-value** |
| --- | --- | --- | --- |
| N | 1,239 | 987 |  |
| Maternal age (years) | 33.6  (33.3, 33.8) | 33.2  (32.6, 33.7) | 0.2 |
| Maternal pre-pregnancy BMI (kg/m^2^) | 24.1  (23.8, 24.3) | 24.1  (23.1, 25.0) | 0.9 |
| Parity | 1.8  (1.7, 1.8) | 1.9  (1.8, 2.0) | 0.05 |
| Male & Female fetuses (n, (% )) | 636 & 588  (52.0 % male) | 219 & 211  (50.9 % male) | 0.7 |
| Smoked & Not Smoked during pregnancy (n (%)) | 43 & 1,182  (3.5 % smoked) | 43 & 387  (10.0 % smoked) | 5.8 x 10^‑7^ |
| Unadjusted birth weight of the baby (kg) | 3.505  (3.474, 3.535) | 3.403  (3.352, 3.454) | 2.8 x 10^‑4^ |
| Birth weight of the baby adjusted for gestational age (kg) | 3.487  (3.461, 3.512) | 3.444  (3.400, 3.487) | 0.1 |
| Adjusted birth weight of the baby* (kg) | 3.478  (3.452, 3.504) | 3.452  (3.357, 3.546) | 0.5 |

*Adjusted for gestational age at birth, sex, multi-fetal pregnancies, maternal parity, pre-pregnancy BMI and smoking.

**Supplementary Table 2** Characteristics of the women with and without GDM from the Cambridge Baby Growth Study who took part in this study.

| **Characteristic** | **Without GDM** | **With GDM** | **p-value** |
| --- | --- | --- | --- |
| N | 780 | 85 |  |
| Age at Menarche (years) | 12.9  (12.8, 13.0)  (n=762) | 12.8  (12.5, 13.1)  (n=84) | 0.6 |
| Maternal age (years) | 33.4  (33.1, 33.7)  (n=708) | 33.2  (32.3, 34.1)  (n=79) | 0.6 |
| Maternal pre-pregnancy BMI (kg/m^2^) | 23.9  (23.6, 24.2)  (n=700) | 26.4  (25.4, 27.4)  (n=76) | 1.9 x 10^-6^ |
| Parity | 1.7  (1.6, 1.8)  (n=774) | 1.8  (1.7, 2.0)  (n=84) | 0.1 |
| Male & Female fetuses (n, (% )) | 396 & 377  (51.2 % male) | 47 & 36  (56.6 % male) | 0.4 |
| Smoked & Not Smoked during pregnancy (n (%)) | 25 & 748  (3.2 % smoked) | 6 & 77  (7.2 % smoked) | 0.06 |
| Gestational age at the birth of the baby (weeks) | 40.0  (39.9, 40.1)  (n=774) | 39.4  (39.0, 39.7)  (n=83) | 3.0 x 10^-4^ |
| Unadjusted birth weight of the baby (kg) | 3.494  (3.457, 3.530)  (n=772) | 3.660  (3.549, 3.771)  (n=83) | 5.5 x 10^-3^ |
| Birth weight of the baby adjusted for gestational age (kg) | 3.468  (3.436, 3.500)  (n=772) | 3.743  (3.646, 3.840)  (n=83) | 2.0 x 10^-7^ |
| Adjusted birth weight of the baby* (kg) | 3.465  (3.433, 3.497)  (n=698) | 3.724  (3.625, 3.822)  (n=76) | 1.4 x 10^-6^ |

*Adjusted for gestational age at birth, sex, multi-fetal pregnancies, maternal parity, pre-pregnancy BMI and smoking.

**Supplementary Table 3** Characteristics from the OGTTs of the women from the Cambridge Baby Growth Study who took part in this study.

| **Characteristic** | **Values** |
| --- | --- |
| Fasting venous glucose concentrations (mmol/L) | 4.3  (4.3, 4.3)  (n=865) |
| 60 min. venous glucose concentrations (mmol/L) | 6.6  (6.5, 6.7)  (n=855) |
| Fasting plasma insulin concentrations (pmol/L) | 45  (44, 47)  (n=846) |
| 60 min. plasma insulin concentrations (pmol/L) | 351  (338, 364)  (n=838) |
| Fasting plasma C-peptide concentrations (nmol/L) | 0.55  (0.54, 0.57)  (n=749) |
| 60 min. plasma C-peptide concentrations (nmol/L) | 2.54  (2.48, 2.60)  (n=742) |
| Area under the capillary glucose tolerance test curve (mmol.min/L) | 823  (809, 838)  (n=642) |
| (C-Peptide derived) HOMA IR | 3.44  (3.34, 3.55)  (n=749) |
| (C-Peptide derived) HOMA B | 313  (306, 319)  (n=749) |
| Insulinogenic index | 140  (134, 147)  (n=796) |
| Insulin disposition index | 15,092  (14,301, 15,927) |
| C-peptidogenic index | 0.92  (0.88, 0.97)  (n=701) |
| C-peptide disposition index | 0.55  (0.52, 0.58)  (n=701) |

Data are geometric means (95% confidence intervals).
